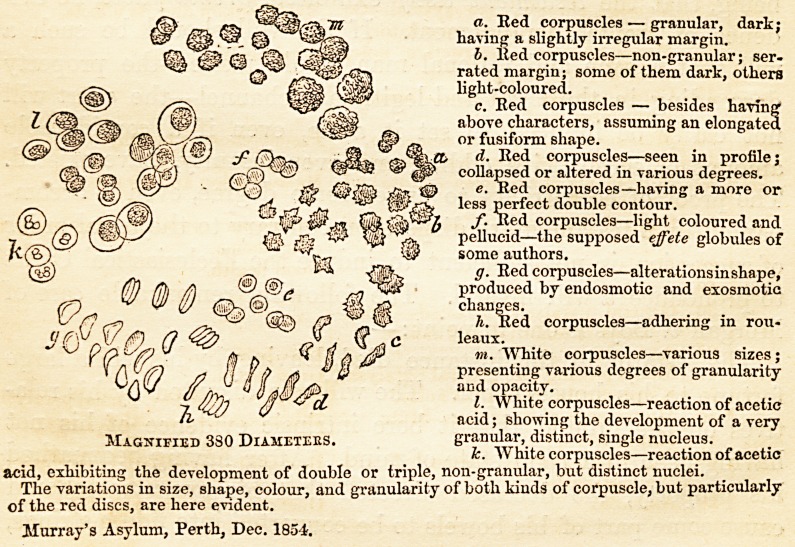# The Histology of the Blood in the Insane

**Published:** 1855-01-01

**Authors:** W. Lauder Lindsay

**Affiliations:** Late Assistant Physician Crichton Royal Institution, Dumfries.


### Art. VII.—THE HISTOLOGY OF THE BLOOD IN THE INSANE.

By W. LAUDER LINDSAY, M.D.,

Late Assistant Physician Crichton Royal Institution, Dumfries.

The subject of the structural alteration of the blood in insanity is one which, so far as I am aware, has hitherto attracted little or no attention in this country, either among psychological physicians in particular or medical observers in general. I hope, however, to show, in the following remarks, that it is one specially worthy of investigation, aided by all the light of modern discoveries in histology, chemistry, and pathology: not, perhaps, as elucidating the mysteries of morbid conditions of the mind, or its organ, the brain, but as powerfully illustrating the laws of general and special pathology. Researches of this nature will tend greatly to break down the unfounded prejudices still existing in the public mind regarding the special nature of insanity, and to propagate, among the profession as well as the public, more correct opinions of the mutual relations of healthy and morbid states of mind and body, and more particularly of the reaction of physical disease on mental phenomena. It will hereby be found that insanity is much more a corporeal disease than is at present believed, or, at least, is more intimately connected with, or inseparable from, various of the ordinary physical diseases to which human flesh is heir.

The following remarks are founded on the results of a microscopical examination of the blood in 236 insane patients, and in thirty-six officers and attendants in the Crichton Royal Institution and Southern Counties Asylum at Dumfries.* These asylums are beautifully situated on the brow of a hill,

* I gladly avail myself of the opportunity of expressing my deep obligations to Dr. Browne, the present superintendent of these asylums, for permitting me to make and record the following observations; and to Mr. Aitken, late house surgeon of the Southern Counties Asylum, for his courtesy and kindness in assisting my investigations among his patients.

which slopes gently towards the Nith: the panorama they command is one of great and varied beauty; though sheltered from certain winds, they are freely exposed to the sea-breeze, which sweeps up Nithsdale from the Solway Firth; the grounds are commodious and attractive; the sanitary arrangements are excellent; and the internal economy is at least equal, if not superior, to that of any similar institutions in the kingdom. The former asylum was built some fifteen years ago, and contains an average number of 120 patients, belonging to the middle and higher classes of society. Many of them have been nurtured amid all the comforts, if not the luxuries and elegancies of life, and are highly educated and accomplished; the others have at least moved in respectable society, and have received all the advantages of modern education. They are still surrounded, so far as personal and general safety and the discipline of a large establishment will allow, with most of the comforts of home: they have frequent or daily open-air exercise, but their occupations and amusements, from their previous education and habits, are *sedentary* and *intramural*. The latter is a model of a pauper asylum, having been recently built, with all the most important modern improvements, under the immediate supervision, and with the advantage of the skilled experience, of Dr. Browne. It contains an average number of 180 patients, chiefly from the neighbouring counties of Dumfries, Kirkcudbright, and Wigton; many of them are not paupers, in the ordinary acceptation of the term, but belong to the middle classes, have moved in good society, and received excellent, some of them university, educations, and have been placed there in consequence of the inability of friends to pay higher rates of board. The majority of the males are engaged daily in *active* and *open-air occupations* and amusements, while the females chiefly engage in needlework in a large commodious work-room.

The two establishments possess an ample staff of officers, attendants, and servants, most of whom, previous to their entering the service of the respective asylums, have been engaged in various rustic occupations, or have worked at various healthy trades in country districts. Many of them—females as well as males—are remarkably tall, athletic, and handsome; most of them are in robust health. A few, however, are not exempt from the cachexies and diseases so common in all ranks of society: various forms of scrofulous disease in the males, and of uterine affections in the females, being the chief morbid conditions.

At the time of my experiments (and speaking generally) the inmates of both asylums were in good physical health. But, in a community of such a size and constituted of such varied elements, and bearing in mind that insanity is rarely, if ever, quite unconnected with bodily disorder, it would have been unique and unnatural had there not existed a considerable amount of the same functional or organic diseases to which the sane are liable. It appears advisable shortly to catalogue the chief physical complications, as well as the classes of mental alienation, in the patients whose blood was examined, in order to place us in a more favourable position for contrasting the structural alterations in the blood corpuscles in the insane and sane, under the same or different circumstances regarding the presence or absence of these physical complications or diseases.

I. Cachexies and general systemic affections:

- Strumous diathesis.

- Hæmorrhagic diathesis.

- Anæmia.

- Plethora.

- Syphilis.

II. Diseases of the skin and cellular tissue:
- Cutaneous eruptions—acné, scabies, psoriasis.
- Carbuncles and boils.
- Erysipelas and erythema.
- Ulcers and abscesses, connected with struma, syphilis, varix, &c.

III. Diseases of the digestive system and alimentary canal:
- Dyspepsia—in all its usual forms.
- Chronic vomiting.
- Obstinate constipation.
- Diarrhœa—simple, chronic, dysenteroid.
- Dysentery—acute, chronic.
- Cholera.

IV. Diseases of the respiratory system:

- Phthisis—especially in its earlier stages.

- Bronchitis.

- Asthma.

- Pneumonia.

- Pleurisy.

- Influenza.

- Pneumo-thorax and hæma-thorax.

V. Diseases of the circulatory system:
- Heart disease—functional and organic.
- Tendency to syncope.
- „ apoplexy.

VI. Diseases of the liver and kidney:
- Hepatitis—chronic.
- Diabetes—mellitus.
- „ insipidus.
- Oxaluria and other morbid conditions of urine.
- Anasarca—renal.

VII. Diseases of the nervous system:
- Paralysis—in various degrees.
- Hysteria, catalepsy.

VIII. Diseases of the genito-urinary system:

- Spermatorrhœa.

- Gonorrhœa—gleet.

IX. Diseases of the uterine system:

- Menorrhagia, amenorrhœa, leucorrhœ

- Polypus.

X. Diseases of the bones and joints:
- Rheumatism.

XI. Diseases of the lymphatic system:
- Bronchocele.

XII. Diseases of the organs of special sense:

- Ophthalmia.

- Otorrhœa.

- Strabismus.

XIII. Surgical diseases:
- Fractures—skull, sternum, ribs.
- Dislocations—tibia.
- Spinal curvature.
- Scrofulous diseases of the bones.
- Hernia.
- Fistula in ano, hæmorrhoids.
- Emphysema of cellular tissue.

The types or phases of insanity in the patients were the following:—

I. Mania: simple or general, acute or chronic, periodic, remittent, &c.

- Religious.

- Erotomania.

- Satyriasis.

- Nymphomania.

- Homicidal.

- Suicidal.

- Kleptomania.

- Pyromania.

- Puerperal.

- Dipsomania.

- Complicated with epilepsy.

- „ „ dementia.

II. Monomania:

| | |
|---|---|
| Religious. | Homicidal. |
| Joyous, sad. | Suspicious. |
| Superstitious. | Proud. |
| Of fear. | Of Discontent. |

III. Melancholia:

| | |
|---|---|
| Religious. | Suicidal. |

IV. Dementia.

V. Amentia.

VI. General paralysis; especially in the early stages.

VII. Moral insanity.

It is scarcely necessary to observe that in none of the patients could any one of the above types or phases be said to exist in a pure and uncomplicated form: they were usually combined in different forms or degrees.

The following numerical table will show concisely the proportional number of patients and attendants in the two asylums, whose blood was examined:

| | | ATTENDANTS. | | PATIENTS. | |
|---|---|---|---|---|---|
| Crichton Institution | Male | 10 | | 61 | |
| | Female | 2 | | 39 | |
| | | | — 12 | | — 100 |
| Southern Counties Asylum | Male | 17 | | 90 | |
| | Female | 7 | | 46 | |
| | | | — 24 | | — 136 |
| | | | 36 | | 236 |
| | | | | | 36 |
| | | | | Total | 272 |

Of the 236 cases in both asylums, the following was the proportion belonging to the great types or classes of mental alienation just enumerated:—

| | PER CENT. |
|---|---|
| Mania | 42.3 |
| „ with epilepsy | 2.7 |
| Monomania | 11.8 |
| Melancholia | 12.9 |
| Dementia | 25.4 |
| Amentia | 1.6 |
| General paralysis | 3.3 |
| | 100.0 |

Of the 36 attendants in both asylums, 75 per cent. were healthy, and 25 were affected with some of the diseases previously referred to.

In these classes of persons, I was afforded an opportunity of—

I. Studying the histology of the blood in the insane of both sexes, of all ages,

*a.* from all parts of Britain, belonging to all classes of the community, whose previous education, habits, and diseases, had been of the most diversified kind.

*b.* In all the more ordinary, as well as in many of the rarer, forms of mental alienation.

*c.* In various forms of insanity, complicated with every kind and degree of physical disease.

*d.* In a limited number of the sane, also variously circumstanced regarding their physical condition.

II. Contrasting the structural condition of the blood in various great
*a.* divisions of the insane, *e.g.*, the rich and pauper insane.
*b.* In various forms of mental alienation, *e.g.*, mania and general paralysis.
*c.* In various physical complications accompanying the same, or different, forms of insanity, *e.g.*, phthisis and cholera.
*d.* In the healthy and the diseased insane.
*e.* „ sane and insane.
*f.* In the sane and insane affected with the same physical diseases.

The blood examined was, in almost all cases, that drawn from the point of some of the fingers by the prick of a needle. In one case, where the patient refused to submit to this slight operation, it was taken from some coagula in a scrofulous abscess of the neck. From the character of the patients, the examination was necessarily superficial and hurried; but the results, though in many points unsatisfactory, were sufficiently distinct to indicate certain general facts regarding the relative condition of the blood in the insane and sane. As a general rule, the insane are extremely bad subjects for such experiments. This applies, of course, in different degrees, to patients labouring under different forms of insanity. They are extremely sensitive, restless, and suspicious of operative interference, even of so slight a nature. Many obstinately refused to allow their fingers to be pricked. Some did so from a firm conviction that a deep-laid conspiracy against their lives or welfare lurked under the cloak of apparently simple experiment; others simply objected to become tools of experiment or amusement; some declined on the plea that in their greatly debilitated condition they could ill afford to spare even a single drop of blood; others lacked courage to submit to the operation; some demanded full explanations of the motives which led to my making the singular request of allowing their finger to be pricked by a needle; in others this formed the key-note of their delusions, delirium, or vituperation, for days or weeks after the experiment was attempted in them. On the other hand, many, who could not appreciate the objects of experiment, submitted cheerfully, merely from a wish to please their medical attendant; others—chiefly cases of confirmed dementia or of deep lethargy—were perfectly passive, freely permitting any kind or amount of experimentation; some presented their fingers, under the impression that, from the single drop of blood, the state of their constitution, the chances of cure, and the period of their removal, could infallibly be predicted; others from curiosity to see the appearance which their own blood, or that of their companions, presented under a microscope; many, especially of the educated classes, comprehending at once the objects of experiment, cheerfully submitted, and evinced the liveliest interest in the microscopical appearances, which, in all cases where the patient was in a condition to appreciate them, were demonstrated and explained; some carried this laudable curiosity to a great extent, begging most earnestly not only to see their own blood at different periods of the day, but that of fellow-patients and attendants, evidently strongly impressed with the belief that between their own blood and that of companions who exhibited most different traits of character or conduct, or between that of insane patients and sane attendants, there should exist a perceptible difference. On various occasions, I was obliged to demonstrate the condition of my own blood under the microscope, to satisfy the curiosity thus awakened. There was a marked difference between the two asylums in the readiness with which both patients and attendants submitted to experiment. In the Crichton Institution, a much larger proportion submitted, and with greater cheerfulness and readiness, than in the Southern Counties Asylum, where a great amount of persuasion and explanation was frequently necessary. It may, at first sight, appear surprising that the experiment should have been more successful among the rich than the poor insane,—among persons of refined habits, and many of them of delicate constitutions, than among rough, hardy artisans and field-labourers. The difference I attribute entirely to the difference in the *education* of the respective classes; to which, also, I attribute the fact that the patients in the Crichton Institution submitted more readily and cheerfully than the attendants. It is noteworthy, moreover, that, among the higher class patients, a much larger proportion of ladies than gentlemen offered themselves as the subjects of experiment. The cause of this difference appeared to be that curiosity strongly predominated in the former. They evinced great anxiety to know the difference in the condition of the blood between the sane and insane, the diseased and healthy. The superior courage with which they bore the operation, simple as it was, cannot, however, be explained on the same ground. The classes of cases most readily experimented on were amentia, confirmed dementia, melancholia, and general paralysis; those least readily, mania and monomania. The microscope used in the investigations was one of Nachet's (of Paris); the magnifying power varied from 180 to 380 diameters,—most frequently the former. In consequence of the difficulty to which I have already adverted,—of prosecuting such researches among the insane,—I was unable, in the majority of cases, to examine the blood of the same individual more frequently than once; and as I was obliged to do so when favourable circumstances in each individual case presented themselves, my examinations were made at irregular periods of the day. I was thus prevented from making other than a qualitative and rough examination,—from ascertaining the variations in the condition of the blood according to the period of the day (in connexion with the digestion of food, &c.), sex, age, and type of disease, mental and bodily, and from accumulating similar data on which to found general deductions,—which I should, under more favourable circumstances, have endeavoured to do.

When drawn, the blood, in the majority of cases, presented to the naked eye the characters of healthy blood; but in a certain number of cases it varied in,—

1. Colour, granularity, and dulness; 2. Density or consistence;
3. Coagulability; 4. Readiness of its flow;
5. Rapidity of separation of the red globules and fibrin; and
6. The apparent relative amounts of serum and crassamentum.

It sometimes possessed a bright orange-red tint, or presented various shades of crimson, purple, or brick-red. In some cases, there was little or no tendency to coagulation, the crassamentum being very loose and imperfect, or the serum maintaining a distinctly red colour, the crassamentum absent, or nearly so, and the red globules forming a pulverulent or granular basis of a dull brownish-red colour. In this condition it resembled blood drawn from the dead body twelve or eighteen hours after death, in which the fibrin appears either to be deficient in quantity, or to have been retained in the form of coagula in some of the vessels. In many of these cases the blood appeared to be very fluid: in others it was as decidedly the reverse. Sometimes the red discs rapidly became agglomerated into rouleaux, forming distinct red streaks or striæ in the straw-coloured serum; in other cases, not the least trace of this phenomenon was visible. Considerable variety also existed regarding the readiness with which blood was drawn, and the amount thereof; the depth of the needle-wound, and the other circumstances of experiment, being, as nearly as possible, in all cases, the same. This was doubtless due, in great measure, to variations in the thickness of the skin and vascularity of the points of the fingers in the rich and poor insane, to anæmia in some cases and plethora in others, and similar circumstances, which do not immediately or necessarily enter into the subject of the present remarks.

In a large proportion of cases, both kinds of corpuscles—red and white—presented their normal characters under the microscope, but in many there existed certain deviations therefrom, which I shall briefly detail under the following heads:—

I. Variations in relative number.
II. " colour, granularity, opacity.
III. " size.
IV. " form.
V. " tendency to agglomerate.
VI. " reaction of acetic acid.

I.—*Red Corpuscles.**—*Number.*—I had no means of accurately estimating the relative proportion, compared with the normal standard, present in each or any case, but from the large proportion, or excess, of white corpuscles found in many cases, and from the general appearance of the blood, it is highly probable that there was frequently a more or less marked diminution in the relative number of red discs, especially in certain cases of anæmia and chronic debilitating disease.

*Colour.*—They were sometimes very dark, chiefly when of small size, granular on the surface, and irregular in shape; more rarely, and chiefly when of large size, they were light, and of a pale yellow colour. In the latter cases, the central depression was frequently very indistinct, or altogether absent; and in these circumstances the corpuscles resembled pellucid globules. By some observers (*e.g.*, Virchow) pale bodies, having these or similar characters, are regarded as defunct blood discs incapable of performing the functions peculiar thereto, and in particular of acting as absorbers and carriers of oxygen to the tissues. In support of this opinion, it has been lately found that frogs, whose liver had been excised, lost the power of respiring carbonic acid and of absorbing oxygen in proportion as the pale clouded globules increased in number.† Granularity was most marked when the corpuscles were of their normal size, or less. When the light-coloured and larger corpuscles were granular, they were almost indistinguishable from the white corpuscles. The granules were sometimes aggregated in such a way as to resemble nuclei. Many of the light-coloured globules, when thickly agglomerated in masses, became much darker, showing that the variations in colour, in many cases, depended, to a great extent, on the effects of light.

*Form.*—Sometimes they were irregularly angular, presenting various resemblances to squares, rhombs, or triangles; by irregular bulgings they became cymbiform, ellipsoid, spheroid, globular, and curved in various ways; and by elongation they assumed fusiform, pyriform, caudate, and staff-shaped appearances. Sometimes they resembled grains of wheat, having a central raphé—apparently a line of puckering. The margin frequently presented a notched or serrated appearance, due, seemingly, to collapse of the walls. This was most frequently noticed in discs which were at the same time small and granular; it existed rarely in those of unusual size, and it was seldom found in those having an elongated form. The central depression was marked in various degrees; sometimes, as in the embryonic blood corpuscle, it was absent. Occasionally, the circumference of the discs presented the appearance of a more or less perfect double contour. I have noticed appearances similar to some of the above in the blood of cholera.‡ Most of these forms have been described by various observers as indicative

* As in many cases blood was obtained in so small quantity as to necessitate dilution, and in order to insure uniformity in the results, water was, in all cases, added under the microscope.

† Moleschott's Experiments. Müller's Archiv, or British and Foreign Medico-Chirurgical Review, Oct. 1854.

‡ Edinburgh Monthly Journal of Medical Science, Aug. 1854; p. 133.

of the decay and death of the blood corpuscles; and they regard such a condition as of great pathological importance, bearing on the etiology and pathology of various diseases. These modifications of the common red disc, the supposed products of decay or disorganization in debilitated constitutions, appear to be produced by endosmotic and exosmotic changes dependent on the loss of equilibrium or affinity between the corpuscles and the liquor sanguinis. Other authors assert that many of the above forms, though closely resembling the modifications resulting from incipient or advanced disintegration, are essentially distinct therefrom; but have, nevertheless, an equally significant pathological importance. Frequently I noticed that, while a comparatively few corpuscles in a particular part or parts of the field of the microscope were thus altered in character, the remainder were perfectly normal in appearance. This renders it possible, or even probable, that many of the changes in the appearance of the red discs may have been produced by physical causes operating at the moment, *e.g.*, unequal pressure between the glass-slides, unequal dilution with water, &c.

*Size.*—I have already mentioned incidentally the variation in size. In some cases they were so small and light coloured as to resemble oil globules; in others they equalled or exceeded in size the white corpuscles.

*Tendency to unite into Rouleaux.*—Instead of rouleaux, the corpuscles often became aggregated into irregular masses, having a dark colour, from their density; at other times there appeared to be no tendency to aggregation of any kind. There was also considerable variation in the rapidity with which such aggregations, whether in rouleaux or irregular masses, broke up or became dissolved.

*Reaction of Acetic Acid.*—No abnormal peculiarities were observed.

II. *White Corpuscles.—Relative number.*—In a comparatively large proportion of cases they were present in excess; in some cases in very marked excess. In many cases the excess may have been only apparent, and really due to deficiency of the red corpuscles in anæmic debilitated patients, labouring under chronic and exhausting affections. In most of these cases they separated gradually from the red discs, and floated to the side of the field, where they appeared in groups of different sizes; they were seldom noticed adhering in any way to each other. This grouping appeared variously due, in different cases, to their lighter specific gravity, whereby they floated out from among the red corpuscles, or to their extrusion from, or repulsion by, the red, while in progress of agglomeration into rouleaux or masses.

It is necessary here to mention that I took no means of estimating quantitatively or accurately the proportion of white to the red corpuscles. I merely judged qualitatively, or in a general sense, of the normal or abnormal relation of these two kinds of corpuscle by comparing the microscopical condition of the blood in the sane and insane, healthy and diseased, persons who were the subjects of experiment. This mode of investigation was of course open to great inaccuracies and fallacies; but it will be found sufficient for arriving at the general results, which it is my object to enunciate. There is no good plan, of easy applicability, for estimating the relative numbers of red and white blood corpuscles in a given specimen of blood. Most elaborate micrometrical enumerations have been tried by Vierordt and other continental microscopists; but this means is so tedious and difficult as to be practically impossible. Professor Bennett has suggested that the best means to form an estimate is to observe the spaces or meshes between the rouleaux or aggregate masses of the red discs. But this mode of procedure is very fallacious. I have repeatedly failed to detect a single white corpuscle in this way, when I knew they existed in considerable numbers, and even in excess, and where I have subsequently succeeded in proving their presence by floating them out in water. Observers are very much divided as to what constitutes the normal proportion of the white to the red corpuscles. For some time it has been generally held to be one white to every eight or ten red: but late experiments on the continent seem to prove that this is very erroneous.* The importance of the subject in connexion with these experiments will, I hope, be a sufficient excuse for very briefly mentioning a few of the results referred to. Donders and Moleschott state the average proportion to be 1 to 373. They found that in persons between two and a half and twelve years of age, the average proportion was 1 to 226; between thirty and fifty years, 1 to 346; in old men between sixty and eighty, 1 to 381; in females, after menstruation, 1 to 247; in females who had not menstruated, 1 to 405; and in pregnant women, 1 to 281. The white corpuscles increased after food, especially if rich in albumen, and diminished by fasting; they were increased also during menstruation and pregnancy.

*Granularity and Opacity* varied considerably; they were most marked where the corpuscles were not increased in size, or were smaller than normal.

*Size.*—Sometimes they resembled the red corpuscles in size; at other times they attained two or three times their normal bulk; in the latter case they were very pellucid, non-granular, and delicate.

*Form.*—Irregularities in the outline were comparatively seldom met with, and were more probably temporary and due to physical causes in operation during the microscopical examination, than permanent or structural changes. A large granular opaque nucleus was sometimes visible without the aid of acetic acid. It usually occupied nearly the whole cell; sometimes it was central, at other times more or less parietal; in the latter case the cell wall resembled a delicate vesicle or veil enveloping the nucleus.

*Reaction of Acetic Acid.*—This reagent usually rendered evident a large, granular, simple nucleus, or a double or triple nucleus, which was much smaller and seldom granular, though frequently opaque. The cell wall usually became very distinct, and sometimes swelled to a great extent round the nucleus. Occasionally the nucleus was as, or even more, distinctly visible before the addition of the reagent. Where the nucleus was visible on the simple addition of water, acetic acid generally rendered it only more granular and distinct. Where the double or triple nucleus was developed, the corpuscles closely resembled, and could not have been distinguished from pus cells. This condition of nucleus was chiefly noticed in small-sized corpuscles; the larger, granular, single nucleus in those of larger size. Sometimes the supposed white corpuscle proved, on the addition of acetic acid, to be only the nucleus round which the cell wall was now developed as a very delicate pellucid vesicle.

The alterations which I have above shortly described were much more common among the inmates of the Crichton Institution than those of the Southern Counties Asylum. This is attributable, doubtless, not only to the influence of previous education and habits on the constitution of the patients respectively, but also to the essential difference in the occupation and amusements of the two classes; their passive, sedentary nature in the one, and their active, open-air character in the other. These conditions of the blood were not confined to the insane, for they occurred, to a less extent, however, in the sane. Nor did they appear to bear any relation, in kind or degree, to the type or class of mental alienation; but a connexion was traceable, both in sane and insane, with physical disease.

In estimating, however, the value of such structural changes in the blood in connexion with mental or physical disease in the insane, it is important to bear in mind the following facts *inter alia*. Many, if not all, of the above conditions have been found in other diseases; and it is probable they exist in many bodily states, which are not usually classified as distinct diseases. Variations in size of the corpuscles are known to be comparatively common in health as well as

* Donders and Moleschott. Schmidt's Jahrbuch, No. 6, 1854, or British and Foreign Medico-Chirurgical Review, Oct. 1854.

disease, in persons of all ages and of both sexes. The blood corpuscles very readily assume a great variety of form, temporary or permanent, from simple physical causes—*e.g.*, pressure, or addition of reagents causing endosmotic and exosmotic changes. The red corpuscles are well known to become wrinkled or puckered, and tuberculated or granular, after removal from the body and exposure. Changes in form and colour are frequently produced by the indirect action of medicinal agents which have been received into the system through the medium of the lungs or stomach, or by their direct application to the blood itself. Both white and red corpuscles are increased or diminished in number in many diseases; an increase or decrease of the one, however, may be merely apparent, and due to the decrease or increase of the other. A gradual transition of the red into the white corpuscles has repeatedly been traced in various affections; the red become granular, light coloured, and enlarged; and the white become flattened, non-granular and more opaque. The granularity and irregularity of the margin in the red discs has been variously attributed to puckering from simple desiccation; to the accumulation or adhesion of minute bubbles of common air or gases contained or developed in the blood; or to the adhesion of particles of fibrin.

These and similar considerations, which it is unnecessary here further to specify, are sufficient to indicate the fallacies and mistakes into which we are apt to fall in the investigation of such a subject. My observations have not been sufficiently extensive or minute to enable me to arrive at any very new or valuable results; still my present object shall have been fully answered if I can succeed in inducing observers, of greater experience and larger opportunities, to prosecute researches which I have but crudely begun.

I have appended a few tables of cases illustrative of the facts and fallacies above specified; they are interesting, as much on account of their negative as their positive evidence.

The following is a *resumé* of the chief general conclusions or results at which my experiments appear to warrant me in arriving—viz.:

I. That the blood of the insane varies considerably in
 - *a.* Colour, granularity, and dulness;
 - *b.* Density or consistence;
 - *c.* Coagulability;
 - *d.* Relative proportion of serum, fibrin, and globules;
 - *e.* The tendency of the red discs to agglomerate;
 - *f.* Rapidity, readiness, and amount of the flow.

II. That the *red discs* vary in *a.* size, *b.* form, *c.* colour, *d.* number, *e.* tendency to agglomerate.

III. That the *white globules* vary in *a.* size, *b.* form, *c.* granularity, *d.* number, *e.* reaction of acetic acid.

IV. That, in the blood of the insane, a *leucocythemic condition* frequently exists.

V. That, in many cases, this condition may be more apparent than real, and due to a *deficiency in the amount of red discs.*

VI. That there is no fixed relation between the kind or intensity of the above conditions, and the various forms or phases of mental alienation.

VII. That there is, however, a certain relation between these conditions and the physical complications of mental alienation.

VIII. That these conditions are not peculiar to the insane, but occur in the sane, under similar circumstances of physical disease.

IX. That the blood is more altered in the insane than the sane, chiefly in proportion as anæmia, struma, and other physical states, are more common in them.

X. That, contrasting the condition of the blood in the rich insane, with that in the poor insane, it is deteriorated, more frequently and to a much greater extent in the former.

XI. That this is due, in great measure, to the essential difference in the education and habits in the respective classes: to the predominance of mental over physical culture in the higher classes; and to the predominance of physical over mental exercise in the labouring classes.

XII. That, contrasting the condition of the blood in various forms of mental alienation, no alterations can be considered peculiar to, or frequent in, any one of these forms.

XIII. That contrasting the blood of the insane with that of the sane, any structural alteration in either class is usually due to physical disease.

XIV. That the physical conditions or diseases, both in sane and insane, in which the above structural alterations most frequently occur, are debilitated states of the system and general vitiation of the blood, resulting from long-continued and exhausting diseases, *e.g.*, anæmia resulting from phthisis, menorrhagia, or intestinal diseases.

TABLE I.

Cases illustrative of alteration of the blood-corpuscles, *in connexion with Physical Disease* in the *Insane.*

| Sex. | Age. | Phase of Insanity. | Nature of Physical Disease. | Condition of Blood-corpuscles. |
|---|---|---|---|---|
| M. | 47 | General paralysis, epilepsy — Monomania of riches, kleptomania, mutilator. *Died.* | Anæmia, diarrhœa, dysentery. Said to have had enteritis. | Great increase of *white.* |
| ... | 50 | Homicidal mania, dementia — Occasional abstinence. | Leucophlegmasia, dyspepsia. | Slight increase of *white.* *Red*—small, granular, irregular margin. |
| ... | 36 | Dipsomania, partial dementia. | Delirium tremens, dyspepsia, chronic hepatitis, hypochondriasis. | *White*—slight increase. *Red*—dark, granular, irregular margin; some have the appearance of a double contour. |
| ... | 28 | Confirmed dementia—Functions almost vegetative, dirty and degraded habits. | Struma, tendency to syncope and erysipelas, anæmia. | *White*—slight increase. |
| ... | 28 | Mania, religious and erotic, strong hereditary taint. | Old fracture of skull, struma. | *White*—slight increase, small and very granular. |
| ... | 40 | Confirmed dementia. | Renal anasarca. | *Red*—irregular in shape. |
| ... | 35 | Chronic mania, with epilepsy. | Tendency to erysipelas. | *White*—slight increase. |
| | 19 | Acute [recent] mania, 1st attack. | Phthisis, anæmia, great emaciation and debility, chronic diarrhœa [dysenteroid]. | ,, great increase. |
| ... | 45 | General paralysis, 1st stage, dementia—Monomania of riches. | Pneumonia, fracture of ribs, cutaneous emphysema, diarrhœa, anæmia. *Died.* | ,, slight increase. |
| ... | 30 | Acute mania, with epilepsy. | Anæmia, diarrhœa, debauchery, dissipation. | *White*—increased, small, indistinct. |
| ... | 50 | Monomania of pride. | Syphilis. | *Red*—alteration of shape. |
| ... | 40 | Chronic mania, dementia. | Scrofulous ulcers and abscesses, anæmia. | ,, ,, ,, |

TABLE I.—(*continued.*)

| Sex. | Age. | Phase of Insanity. | Nature of Physical Disease. | Condition of Blood-corpuscles. |
|---|---|---|---|---|
| M. | 50 | Chronic mania, dementia. | Bilious attacks, diarrhœa. | *White*—increased. |
| ... | 45 | Confirmed dementia. | Cutaneous eruptions, ulcers, strabismus. | ,, ,, *Red*—indistinct, altered in shape. |
| ... | 40 | ,, ,, | Dyspepsia, chronic vomiting. | *Red*—irregular in margin. |
| F. | 60 | Senile dementia. | Paralysis—Blind. | *Red*—irregular in mar-margin, granular. |
| ... | 35 | Confirmed dementia. | Scrofulous spinal disease, great distortion. | ,, ,, |
| ... | 47 | Melancholia, religious, suicidal — Dirty and degraded. Hereditary taint. | Anæmia, hypochondriasis. | *Red*—light in colour, agglomerated in irregular masses. |
| ... | 45 | Confirmed melancholia —Vanity, occasional abstinence. | Anæmia, dyspepsia [marked by frequent vomiting], oxaluria, intemperance. | *White*—great increase, small, granular. |
| ... | 35 | Melancholia, paroxysmal mania — Occasional abstinence. | Strumous disease of tarsus and metatarsus, anæmia, emaciation. *Died.* | *Red*—altered in shape, agglomerated in masses. |
| ... | 35 | Chronic mania—Indecent, degraded, very incoherent. | Anæmia, cholera. *Died.* | *White*—increased, small, granular. |
| ... | 30 | Chronic dementia—Mute. | Strumous ulcers and abscesses. | *White*—increased, small, granular. |
| ... | 35 | Puerperal mania. | Struma, anæmia. | *Red*—granular. |
| ... | 40 | Partial dementia. | Chronic acné [inveterate]. | *White*—increased. |
| ... | 35 | Melancholia, paroxysmal mania.—Convalescent. | Struma, dyspepsia, bilious attacks, anæmia. | *Red*—altered in shape, granular. |
| ... | 28 | Melancholia, paroxysmal mania — Dirty, degraded, indecent. | Phthisis [vicarious]. | *Red*—altered in shape, granular. |
| ... | 55 | Melancholia, mania. | Strumous abscesses, bronchocele, anæmia. | *Red*—altered in shape, irregular in margin. |
| ... | 40 | Monomania [simple]. | Anæmia. | *Red*—altered in shape, irregular in margin. |
| ... | 40 | Chronic mania. | ,, | *White*—increased, large, granular, irregular in shape. |
| ... | 64 | Melancholia. | Chronic diarrhœa. | *White*—increased, altered in size and shape, irregular in margin. |
| ... | 35 | Chronic mania. | Tendency to dysentery. | *Red*—altered in shape. |
| ... | 25 | Monomania of pride, erotic, mania paroxysmal. | Amenorrhœa, dyspepsia. | *White*—increased, small. *Red*—elongated, light in colour, agglomerated in irregular masses. |

In the above Table, it will be observed that an abnormal condition of both kinds of blood discs sometimes occurred in the same individual; that the morbid condition of the red discs was most frequently alteration in form; that of the white globules, simple increase in number; that in both the cases where there was a marked excess of white globules there was a great amount of physical disease, as well as a severe type of mental alienation; and that the same structural alterations occurred in the most opposite and varied forms of insanity and its physical complications.

TABLE II.

Cases illustrative of alteration of the blood corpuscles, *without the presence of marked Physical Disease,* in the *Insane.*

| Sex. | Age. | Phase of Insanity. | Physical Condition. | Condition of Blood-corpuscles. |
|---|---|---|---|---|
| M. | 45 | Chronic mania, dementia, partial—Delusions. | Robust health, plethoric, occasional epistaxis. | *White* — increased; small, granular, hazy; very smooth margin. |
| ... | 26 | Dementia, partial, congenital. | Robust health, florid complexion. | *Red*—granular, irregular in margin. |
| ... | 26 | Dementia, partial, congenital. | Robust health, florid complexion. | *Red*—altered in shape. |
| ... | 28 | Melancholia—Religious. | Healthy, though of delicate build. | *Red*—altered in shape, margin irregular. |
| ... | 80 | Confirmed dementia, [senile]. | Good health. | *Red* and *white*—altered in shape, margin and granularity. |
| ... | 45 | Chronic mania. | ,, ,, | *White*—increased. |
| ... | 40 | General paralysis. | ,, ,, | ,, ,, |
| ... | 64 | Chronic mania—Delusions, vanity. | ,, ,, | ,, ,, |
| ... | 40 | Chronic mania. | ,, ,, | *White*—increased, very granular and distinct. |
| ... | 30 | Melancholia. | ,, ,, | *White* — increased and altered in shape. |
| ... | 35 | Dementia. | ,, ,, | *Red* and *white* altered in shape. |
| ... | 30 | Monomania—Religious. | ,, ,, | *White*—increased. |
| ... | 35 | Dementia. | ,, ,, tendency to obesity. | *Red* — granular, hazy, irregular in margin. |
| ... | 35 | Mania—Delusions. | Good health, robust, active. | *Red*—small, granular, margin serrated. |
| ... | 40 | Chronic mania, dementia—Delusions. | Good health. | *White*—increased. |
| ... | 40 | Monomania of suspicion. | ,, ,, | ,, ,, |
| ... | 35 | Dementia, mania paroxysmal—Hallucinations, dirty, degraded, indecent, mutilator. | ,, ,, | *Red*—altered much in shape, [elongated, fusiform, &c.] light in colour; agglomerated in masses. |
| ... | 50 | Mania, dementia. | ,, ,, | *White*—very granular, smooth margin, distinct. *Red*—altered in shape. |
| ... | 55 | Melancholia — Abstinent, [requiring artificial feeding]. | ,, ,, | *White*—increased, small, smooth in outline. |
| ... | 50 | Mania—Religious. | ,, ,, | *White*—increased. |

TABLE II.—(*continued.*)

| Sex. | Age. | Phase of Insanity. | Physical Condition. | Condition of Blood-corpuscles. |
|---|---|---|---|---|
| F. | 45 | Monomania—Religious mania [nocturnal paroxysms.] | Good health. | *Red*—altered in shape, size, colour; margin irregular. *White*—increased, distinctly nucleated, very granular; resemble pus cells in reaction of acetic acid; altered in size. |
| ... | 45 | Dipsomania—Vanity. | Good health, very stout. | *Red*—altered in shape; granular. |
| ... | 45 | Confirmed dementia—Dirty and degraded to an extreme degree. | ,, ,, | *White* — increased, small. |
| ... | 45 | Kleptomania, paroxysmal mania. | ,, ,, | *White*—increased, very granular. *Red*—altered in shape, granular. |
| ... | 45 | Melancholia—1st attack. | ,, ,, | *White*—increased, altered in shape. |
| ... | 35 | Melancholia — Abstinence [requiring artificial feeding.] | ,, ,, | *White* — increased, large, granular. |
| ... | 45 | Mania—Suicidal, homicidal, impulsive. | ,, ,, | *Red*—irregular in margin, granular, dark, hazy. |
| ... | 35 | Mania—Pride. | ,, ,, | *White*—increased, granular, distinct. |
| ... | 45 | Melancholia. | ,, ,, | *Red*—altered in shape. |

TABLE III.

Cases illustrative of the *presence of decided Physical Disease* in the *Insane*, *without* any abnormal alteration of the blood-corpuscles.

| Sex. | Age. | Phase of Insanity. | Character of Physical Disease. |
|---|---|---|---|
| M. | 40 | General paralysis, recurrent mania—Monomania of riches. | Partial paralysis, phthisis, masturbation, debauchery, anæmia. |
| ... | 48 | Senile dementia. | Anæmia, emaciation, constipation. |
| ... | 50 | Dementia, hereditary taint—Delusions. | Phthisis, dyspepsia, anæmia. |
| ... | 28 | Dementia, partial—Mute. | Struma, anæmia. |
| ... | 40 | General paralysis—monomania of riches. | Paralysis, partial, spinal disease; plethora capitis. |
| ... | 50 | Monomania. | Old fracture of skull, tendency to carbuncles. |
| ... | 45 | Monomania. | Old fracture of skull. |
| ... | 30 | ,, of ambition. | Diabetes. |
| ... | 45 | Dementia. | Struma. |
| ... | 30 | Chronic mania. | Ulcers, tendency to erysipelas. |
| ... | 40 | Mania, erotic. | Syphilis. |
| ... | 65 | ,, paroxysmal. | Anæmia, emaciation, senile debility. |
| ... | 40 | ,, | Strumous abscesses. |

TABLE III.—(*continued.*)

| Sex. | Age. | Phase of Insanity. | Character of Physical Disease. |
|---|---|---|---|
| F. | 35 | Melancholia. | Scabies, tendency to erysipelas [traumatic]. |
| ... | 64 | „ | Chronic diarrhœa, anæmia, emaciation. |
| ... | 35 | Mania. | Frequent attacks of dysentery. |
| ... | 40 | „ paroxysmal. | „ „ bronchitis, menorrhagia and dysentery. |
| ... | 40 | Monomania of pride—melancholia. | Dyspepsia, cutaneous eruptions. |
| ... | 23 | Dementia. | Strumous ophthalmia. |
| ... | 28 | Mania ferox, paroxysmal. | Chronic vomiting, angina, anæmia. |
| ... | 20 | Mania, religious—Dirty, degraded; hereditary taint; melancholia. | Amenorrhœa, hypochondriasis, anæmia. |
| ... | 45 | Mania, religious—Delusions, paroxysms of violence. | Strumous abscesses, chronic pleurisy, anæmia. |
| ... | 40 | Mania—epilepsy. | Frequent attacks of dysentery. |
| ... | 40 | „ chronic. | Menorrhagia. |
| ... | 40 | „ „ | Varicose ulcers. |

TABLE IV.

Cases illustrative of the *presence, in the Insane, of great mental impairment*-accompanied or not by physical complications, *without* any abnormal altera, tion of the blood-corpuscles.

| Sex. | Age. | Phase of Insanity. | Physical Condition. |
|---|---|---|---|
| M. | 45 | General paralysis, 1st stage, mania, chronic, paroxysmal—Mutilator, dirty. | Hepatic disorders, sanguineous tumours of the ear. |
| ... | 28 | Mania, chronic, paroxysmal—Occasionally abstinent, mutilator. | Masturbation and its effects. |
| ... | 35 | Mania, chronic—Destructive, noisy. | Good health. |
| ... | 40 | General paralysis, 2nd stage. | „ „ |
| ... | 45 | Chronic mania, dementia—Occasionally abstinent. | „ „ |
| ... | 30 | Monomania of pride, dementia. | Pseudo-chorea; healthy. |
|  | 40 | „ of suspicion, dementia. | „ „ „ |
| ... | 40 | Monomania of suspicion—Mute; advanced dementia. | Masturbation and its effects. |
| ... | 50 | General paralysis, 1st stage. *Died.* | Cutaneous eruptions, tendency to erythema. |
| ... | 35 | Mania, epilepsy. | Good health. |
| ... | 40 | „ „ | „ „ |
| ... | 50 | General paralysis, arrested—Monomania of ambition and riches. | „ „ |
| ... | 25 | Amentia. | Diarrhœa. |
| ... | 40 | Mania passing into general paralysis. | Good health. |
| ... | 70 | Kleptomania, mania—Vanity. | Apoplexy, epilepsy, partial paralysis. |
| ... | 40 | Monomania of suspicion—Mute. | Intemperance; healthy. |
| ... | 40 | Mania ferox. | Formerly a prostitute. |
| ... | 40 | „ paroxysmal, connected with menstruation. | Bronchitis—tendency to dysentery. |
| ... | 20 | Amentia. | Occasional diarrhœa. |

TABLE V.

Cases illustrative of alteration of the blood-corpuscles, *in connexion with Physical Disease*, in the *Sane.*

| Sex. | Age. | Physical Condition. | Condition of Blood-corpuscles. |
|---|---|---|---|
| M. | 24 | Anæmia, sallow, emaciated. | *White*—increased. |
| F. | 40 | Phthisis, menorrhagia, leucorrhœa, chronic hepatitis, anæmia, debility, and emaciation. | ,, ,, |
| ... | 30 | Dyspepsia, menstrual irregularities, anæmia. | ,, ,, |
| ... | 45 | Dyspepsia, leucorrhœa, anæmia. | ,, ,, |

TABLE VI.

Cases illustrative of alteration of the blood-corpuscles, *without* the presence of marked Physical Disease, in the *Sane.*

| Sex. | Age | Physical Condition. | Alteration of Blood-corpuscles. |
|---|---|---|---|
| M. | 35 | Robust health. | *Red*—altered in shape. |
| ... | 30 | Healthy, but of delicate build. | *White*—increased, indistinct. *Red*—granular, margin irregular. |
| ... | 25 | ,, florid complexion. | *White*—increased, small, margin smooth. |
| ... | 30 | Occasional rheumatism. | *Red*—slightly altered in shape. |
| ... | 45 | Good health. | *Red*—granular, margin irregular. |
| ... | 50 | Occasional influenza. | *White*—increased, dark, granular, distinct. |
| ... | 40 | Slight dyspepsia, cutaneous eruptions. | *White*—increased. |
| F. | 28 | Robust health. | *Red*—altered in shape and colour. |
| ... | 35 | Healthy, but occasionally intemperate. | ,, ,, ,, |

MAGNIFIED 380 DIAMETERS.

*a.* Red corpuscles — granular, dark; having a slightly irregular margin.

*b.* Red corpuscles—non-granular; serrated margin; some of them dark, others light-coloured.

*c.* Red corpuscles — besides having above characters, assuming an elongated or fusiform shape.

*d.* Red corpuscles—seen in profile; collapsed or altered in various degrees.

*e.* Red corpuscles—having a more or less perfect double contour.

*f.* Red corpuscles—light coloured and pellucid—the supposed *effete* globules of some authors.

*g.* Red corpuscles—alterations in shape, produced by endosmotic and exosmotic changes.

*h.* Red corpuscles—adhering in rouleaux.

*m.* White corpuscles—various sizes; presenting various degrees of granularity and opacity.

*l.* White corpuscles—reaction of acetic acid; showing the development of a very granular, distinct, single nucleus.

*k.* White corpuscles—reaction of acetic acid, exhibiting the development of double or triple, non-granular, but distinct nuclei.

The variations in size, shape, colour, and granularity of both kinds of corpuscle, but particularly of the red discs, are here evident.

Murray's Asylum, Perth, Dec. 1854.

## Figures and Tables

**Figure f1:**